# Performance Characterisation of the Airvo2^TM^ Nebuliser Adapter in Combination with the Aerogen Solo^TM^ Vibrating Mesh Nebuliser for in Line Aerosol Therapy during High Flow Nasal Oxygen Therapy

**DOI:** 10.3390/pharmaceutics16040565

**Published:** 2024-04-20

**Authors:** Ronan MacLoughlin, Marc Mac Giolla Eain

**Affiliations:** 1 Research and Development, Science and Emerging Technologies, Aerogen Ltd., Galway Business Park, H91 HE94 Galway, Ireland; 2 School of Pharmacy and Biomolecular Science, Royal College of Surgeons in Ireland, D02 YN77 Dublin, Ireland; 3 School of Pharmacy and Pharmaceutical Sciences, Trinity College, D02 PN40 Dublin, Ireland

**Keywords:** vibrating mesh nebuliser, high flow oxygen therapy, aerosol drug delivery, aerosol therapy, adult, paediatric, neonate

## Abstract

High flow oxygen (HFO) therapy is a well-established treatment in respiratory disease. Concurrent aerosol delivery can greatly expediate their recovery. The aim of this work was to complete a comprehensive characterisation of one such HFO therapy system, the Airvo2^TM^, used in combination with the Aerogen Solo^TM^ vibrating mesh nebuliser. Representative adult, infant, and paediatric head models were connected to a breathing simulator via a collection filter placed at the level of the trachea. A tracheostomy interface and nasal cannulas were used to deliver the aerosol. Cannula size and gas flow rate were varied across the full operating range recommended by the manufacturer. The tracheal and emitted doses were quantified via UV-spectrophotometry. The aerosol droplet diameter at the exit of the nares and tracheal interface was measured via cascade impaction. High gas flow rates resulted in low emitted and tracheal doses (%). Nasal cannula size had no significant effect on the tracheal dose (%) available in infant and paediatric models. Higher gas flow rates resulted in smaller aerosol droplets at the exit of the nares and tracheostomy interface. Gas flow rate was found to be the primary parameter affecting aerosol delivery. Thus, gas flow rates should be kept low and where possible, delivered using larger nasal cannulas to maximise aerosol delivery.

## 1. Introduction

Respiratory diseases are amongst the leading causes of morbidity and mortality worldwide. Some of the most common include asthma, pneumonia, chronic obstructive pulmonary disease (COPD), acute respiratory distress syndrome (ARDS), tuberculosis (TB), and cystic fibrosis (CF). According to the WHO, in 2017, 544.9 million people worldwide had a chronic respiratory disease [1]. Treatment can vary depending on severity; however, in most cases some form of respiratory support will be required to alleviate symptoms. The level of support can vary between invasive and non-invasive depending on the condition of the patient; it is delivered via endotracheal tube (ETT), tracheostomy tube (TT), mask, nasal cannula, or mouthpiece. High flow oxygen (HFO) therapy has become an increasingly implemented form of respiratory support as it can be used in both the hospital and homecare setting, improves patient oxygenation, reduces the work of breathing, and allows for concurrent delivery of aerosolised therapeutics for targeted treatment [2,3,4,5].

Numerous bench studies [6,7,8,9,10] and clinical data sets [11,12,13,14,15] have contributed to our understanding of the factors that influence aerosol drug delivery during HFO therapy. In a recent review paper of HFO therapy, Li et al. [16] summarised the current state of the art knowledge in terms of the main factors influencing aerosol drug delivery. These include gas flow rate, gas density, dry versus humidified gas, gas type, delivery interface, position of the nebuliser in the circuit, nebuliser type, gas flow/patient’s inspiratory flow (GF/IF) ratio, open mouth versus closed mouth breathing, and control of aerosol generation during breathing. Of these, the authors concluded that GF/IF ratio, nebuliser type, and position in the circuit were the most important factors in maximising concurrent aerosol delivery. Vibrating mesh nebulisers (VMNs) were found to deliver the greatest quantities of aerosol; they should be placed at the inlet of the humidifier pot and GF/IF should be kept to 0.5 or lower. Bennett et al. had similar findings but concluded that the nebuliser should be placed at the exit of the humidifier pot in order to maximise the aerosol performance of a HFO therapy system [17].

While there are numerous different HFO therapy systems on the market, the most frequently encountered is the Airvo 2^TM^ manufactured by Fisher & Paykel. It can be used in both the critical and homecare environments and can deliver heated, humidified supplemental gas flows suitable for infant, paediatric, and adult patients. It is also one of the few HFO therapy system that has a bespoke nebuliser adapter. The Airvo Neb adapter, the output of a collaboration between Fisher & Paykel and Aerogen, was designed to maximise concurrent aerosol delivery. This adapter integrates the Aerogen Solo^TM^ VMN into the humidifier pot, where the aerosol is entrained into the heated, humidified gas flow exiting the pot. Although this system is frequently cited in the literature, there are conflicting reports on the levels of aerosol delivery at both system and patient level. For example, in studies that have used nasal cannulas, Li et al. [7] reported inhaled doses (%) of 2.7–12%, Murphy et al. [18] 1.37–15.72%, and Bennett et al. [19] up to 28.95% in adults. In similar bench studies for paediatrics, Wang et al. [20] measured tracheal doses (%) between 1.48–3.92%, Réminiac et al. [21] 0.52–4.15%, and Bennett et al. [19] up to 13.54%.

A useful feature of the Airvo2^TM^ system is its compatibility with tracheostomy tubes, which allows it to provide HFO to patients who have bypassed upper airways. Tracheostomy is commonly performed due to either pulmonary insufficiency or due to upper airway obstructions [22]. Similar to HFO therapy via nasal cannula, there have been a number of in-vitro studies that examined means of optimising aerosol therapy delivered through a tracheostomy tube. Nebuliser type and circuit arrangement were found to significantly affect aerosol delivery [10,23,24,25,26,27]. The use of VMN incorporated via T-pieces rather than tracheostomy collars was the recommended option. However, there still exists a risk of accidental decannulation from the additional weight on the tracheostomy tube [28]. The Airvo^2TM^ system, with its bespoke nebuliser adapter and tracheostomy interface, allows a direct connection of the system to the tracheostomy tube, eliminating the need for additional circuit components, potential decannulation, and unwanted dead space. To the best of the authors’ knowledge, neither in vitro nor in vivo data exist on the levels of aerosol delivery possible with this interface. 

The objective of this study was to perform a comprehensive characterisation of the Airvo2^TM^ HFO system delivering concurrent aerosol therapy with its recommended nebuliser, the Aerogen Solo^TM^. This includes both a system and simulated patient characterisation across a comprehensive suite of products, including the previously unreported tracheostomy interface, at the recommended operating ranges. 

## 2. Materials and Methods

### 2.1. High Flow Nasal Oxygen Therapy System

**Figure 1:**
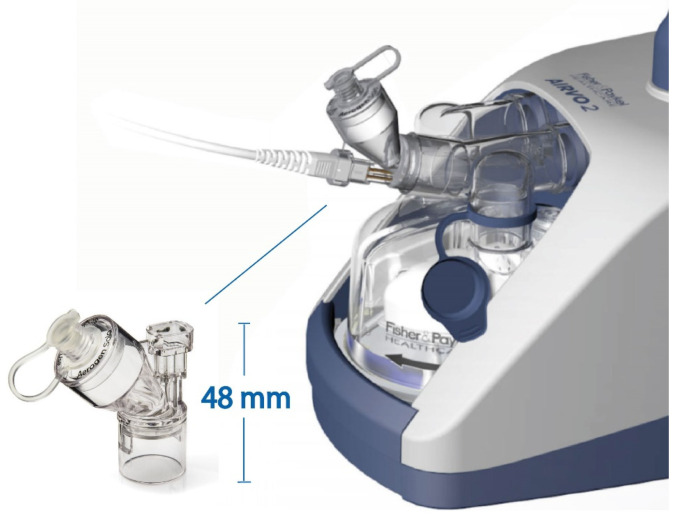
The Fisher & Paykel Airvo 2^TM^ HFO therapy system with integrated Airvo Neb adapter and Aerogen Solo^TM^ vibrating mesh nebuliser (VMN).

The Airvo 2^TM^ HFO therapy system (Fisher & Paykel, Auckland, New Zealand) was used in conjunction with the AirSpiral^TM^ tube and chamber kit and the Airvo Neb adapter (900PT562, Fisher & Paykel, Auckland, New Zealand), as shown in Figure 1. Details of the applicable nasal cannulas and tracheostomy interface used with the Airvo2^TM^ system are listed in Table 1. All interfaces were assessed across their approved gas flow ranges as stated in the Fisher & Paykel user manuals. 

### 2.2. Nebuliser

As per Fisher & Paykel’s user instructions, all testing was completed with the Aerogen Solo^TM^ VMN and the Aerogen Pro-X controller (Aerogen Ltd., Galway, Ireland). The performance characteristics of the VMN were measured by laser diffraction with a Malvern Spraytec particle size analyser (Malvern Instruments, Malvern, UK) and defined in terms of the volume mean diameter (VMD) (Dv50) or average aerosol droplet size. A 5 LPM vacuum flow was implemented through the system, which ensured a laminar flow regime and minimised the artificial growth of aerosol particles due to collisions with other aerosol particles in the plume. The vacuum also ensured that the aerosol plume passed through the laser only once. The centre of the plume was directed through the centre of the laser sheet to increase data acquisition accuracy. Data acquisition was at a rate of 500 Hz. The average aerosol droplet size generated by the VMN was found to be 4.11 ± 0.06 µm at an aerosol output flow rate of 0.45 ± 0.02 mL/min. Given its mode of operation, the Aerogen Solo^TM^ does not introduce flow or pressure to the respiratory circuit, ensuring that the selected gas flow rate is delivered to the patient. Furthermore, dilution of the turbine driven gas flow is avoided; hence, prescribed oxygen levels are maintained, ensuring safer therapy.

### 2.3. System Characterisation

#### 2.3.1. Emitted Dose

**Figure 2:**
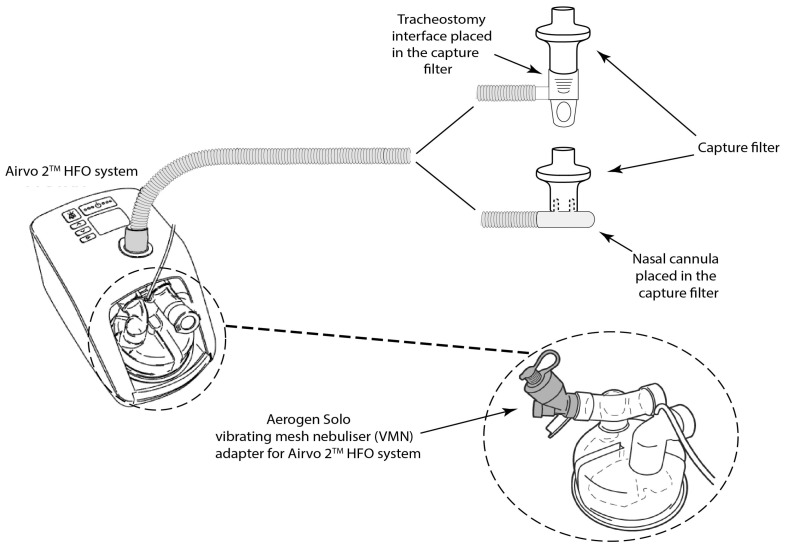
Schematic illustration of the experimental setup used to determine the dose of aerosol emitted at the exit of the different delivery interfaces.

Emitted dose (%) is considered a measure of aerosol transport efficiency through a system, independent of the patient. The key parameters are gas flow rate, nasal cannula size, and system design. Emitted dose (%) was assessed as per Bennett et al. [17]. Figure 2 is a schematic illustration of the setup used, where a capture filter (Respirgard 303, Vyaire, Dublin, Ireland) was placed distal to the exit of the nasal cannula or tracheal interface. As the emitted aerosol exits the interface, it is deposited on the filter, which is free to air on the distal side to allow for the gas flow to pass through. The filter was sealed around the interface on the proximal side to ensure the capture of all aerosols emitted for the relevant interface. A standard dose of 2 mL of 2 mg/mL Albuterol Sulphate (TEVA pharmaceuticals, Waterford, Ireland) was used.

A variety of test combinations representing the various approved combinations of age-appropriate gas flow rate and interface type were assessed and are detailed in Table 2 and Table 3.

#### 2.3.2. Tracheal Dose

**Figure 3:**
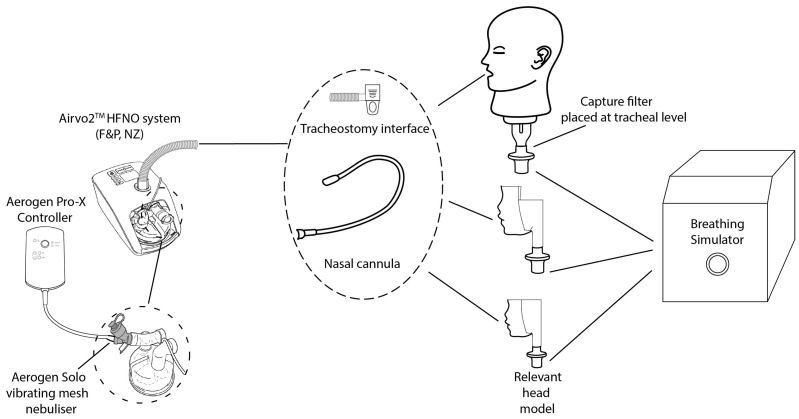
Schematic illustration of the experimental setup used to measure the tracheal dose (%).

The tracheal dose describes the amount of aerosolised drug delivered to the level of the trachea. It is normally expressed as a percentage of the nominal dose placed in the nebuliser’s medication cup (%) and is the typical measure used in bench studies of respiratory drug delivery. Figure 3 illustrates the setup used, where the appropriate interface is placed on the appropriate head model (adult—Life/form^®^ Adult Airway Management Trainer, Nasco Healthcare, Saugerties, NY, USA, paediatric—5-year-old nose throat [19] and infant—(SAINT) [29]. A cuffless 7.5 mm ID inner cannula tracheostomy tube (Shiley, Covidien, Ireland) was used with the tracheostomy interface. The head model was then connected to a breathing simulator (ASL5000, IngMar Medical Inc., Pittsburgh, PA, USA) and the patient-appropriate breath applied. That is to say, those required by regulatory agencies in the characterisation of drug delivery systems, whether they be nebuliser alone, or nebuliser/HFOT system combinations, as shown in Table 4. A capture filter (Respirgard 303, Vyaire, Dublin, Ireland) was placed distal to the trachea, between the head model and the breathing simulator. As with the emitted dose testing, a standard dose of 2 mL of 2 mg/mL Albuterol Sulphate (TEVA pharmaceuticals, Waterford, Ireland) was aerosolised.

The same test combinations assessed for emitted dose were assessed for tracheal dose, representing the various approved combination of age-appropriate gas flow rate (low, mid, and high within each range) and head model and cannula type; these are detailed in Table 2 and Table 3. Where “not tested” is entered, it indicates that this combination of gas flow rate and cannula size was not tested as either the gas flow rate was out of the approved range for the specific cannula, or there was a more appropriate low, mid, or high gas flow tested that better represented the full range for that cannula type.

Both the emitted and tracheal doses (%) were determined by measuring the quantity of aerosol captured on the capture filters, placed at the interface exit or at the tracheal level of the relevant head model. The drug captured on the filter was eluted using 10 mL of deionised water and the mass of drug was then quantified via UV-spectrophotometry at 276 nm. The data was then interpolated on a standard curve of Albuterol Sulphate concentrations of 3.125–200 µg/mL. The results are presented as the average ± standard deviation of the nominal dose placed in the nebuliser’s medication cup.

#### 2.3.3. Droplet Size Characterisation—Mass Median Aerodynamic Diameter (MMAD)

The mass median aerodynamic diameter (MMAD) of the aerosol exiting an interface is considered a predictor of the ultimate location of deposition within the airways. Here, MMAD was characterised at 15 LPM in accordance with EU (Apparatus E, described in EuP 2.9.18 [31]) and US (Apparatus 5, described in USP 1601 [32]) pharmacopeial methods. MMAD and geometric standard deviation (GSD) were calculated using CITDAS (Copley Scientific, Nottingham, UK). A dose of 1.0 mL of 2 mg/mL Albuterol Sulphate (TEVA pharmaceuticals, Waterford, Ireland) was used in order to avoid overloading the impactor plates. MMAD was assessed by placing the different interfaces directly at the entrance of the impactor throat.

The same test combinations assessed for the emitted and tracheal dosages (%) were also assessed as part of the droplet size characterisation, as shown in Table 2 and Table 3.

### 2.4. Statistical Analysis

Each test condition was assessed using 3 individual Aerogen Solo^TM^ nebulisers in triplicate (*n* = 3) for a total of 9 test runs per test combination. Cumulatively, 594 test runs are reported in this study. One-way ANOVA with post-hoc Tukey tests and two-sample *t*-tests were completed to determine significance, which was considered at *p* ≤ 0.05 (Minitab V.19, Minitab LLC, Coventry, UK). 

## 3. Results

To ensure full transparency, the results from each part of this study are included in the Supplementary Materials section of this manuscript. A statistical analysis was performed on the results and the methodology used was documented in Section 2.4. Significance was considered for *p* < 0.05. For each nasal cannula, the effect of supplemental gas flow rate was analysed, and the results of the statistical analysis are presented in the bottom rows of the relevant table. For a given flow, the effect of nasal cannula size was also analysed, presented in the vertical columns in the relevant tables.

### 3.1. Emitted Dose

**Figure 4:**
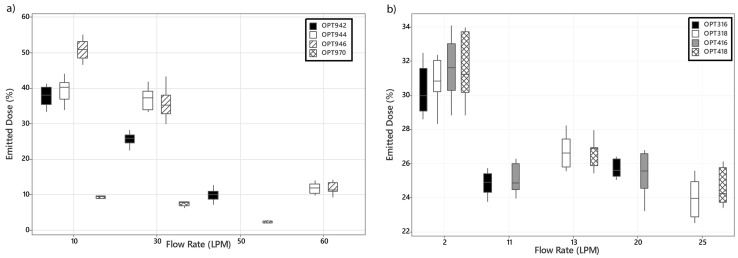
Variations in emitted dose (%) (avg ± SD) with changes in interface, nasal cannula, and supplemental gas flow rate (LPM) for (**a**) adult and (**b**) infant and paediatric models.

Figure 4 presents the variations in the dose of aerosol available at the exit of the interfaces with variations in nasal cannula size and supplemental gas flow rate for (a) adult and (b) infant and paediatrics. Increases in supplemental gas flow rate result in a significant decrease in the quantity of aerosol available at the exit of the nares, *p* < 0.05. In the adult models presented in Figure 4a, at flow rates of 10 and 30 LPM, nasal cannula size had a statistically significant effect on the emitted dose (%) of aerosol, *p* ≤ 0.05, as shown in supplementary data Table S1. However, at 60 LPM, nasal cannula size did not have a significant effect on the emitted dose. At flow rates greater than 2 LPM, cannula size in the infant models OPT316 and OPT416 had no significant effect on the emitted dose, *p* >> 0.05. The opposite was found in the paediatric range of cannula, OPT 318 and OPT418, where the change in cannula size was only found to significantly affect the dose at the exit of the nares at the highest flow rate permissible, 25 LPM, as shown in Figure 4b and supplementary data Table S2. Similarly to the nasal cannula, increases in supplemental gas flow rate resulted in a decrease in the aerosol (%) available at the exit of the tracheostomy interface, 9.38 ± 0.37% at 10 LPM to 2.21 ± 0.31 (%) at 50 LPM, *p* = 0.000.

### 3.2. Tracheal Dose

**Figure 5:**
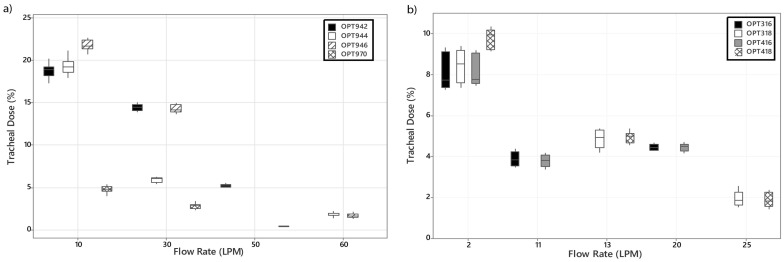
Variations in tracheal dose (%) (avg ± SD) with changes in interface, nasal cannula, and supplemental gas flow rate (LPM) for (**a**) adult and (**b**) infant and paediatric models.

Figure 5 presents the variation in the tracheal dose (%) of aerosol with changes in interface, nasal cannula size, and supplemental gas flow rate (LPM) for both (a) adult and (b) infant and paediatrics. Similar to the emitted dose (%) data shown in Figure 4, increases in supplemental gas flow rate result in decreases in the tracheal dose (%) available in both (a) adult and (b) infant and paediatric models. In the adult model shown in Figure 5a, at supplemental gas flow rates of 10 and 30 LPM, nare sizes OPT942 4.2 mm ID, OPT944 5.1 mm ID, and OPT 946 6.0 mm ID had statistically significant effects on the tracheal dose, *p* ≤ 0.05, supplementary data Table S3. While at 60 LPM, cannula size had no significant effect on the tracheal dose (%), *p* ≥ 0.05. Switching to the infant range, OPT316 and OPT416, as shown in Figure 5b, cannula size changes had no significant effect on the dose of aerosol available at the tracheal level (%) at all flow rates considered, *p* ≥ 0.05, supplementary data Table S4. Comparing the effects of nares diameter and flow rate in the paediatric model, cannula size changes—OPT318 2.9 mm ID and OPT418 3.3 mm ID—only significantly affected the tracheal dose (%) at a flow rate of 2 LPM, *p* = 0.000. Across both size ranges, increases in supplemental gas flow rate significantly reduced the dose of aerosol available at the tracheal level (%), *p* < 0.05. As with the emitted dose (%) data, the aerosol available at the end of the tracheostomy tube significantly decreased with increases in supplemental gas flow rate, 4.75 ± 0.53 (%) at 10 LPM to 0.40 ± 0.08 (%) at 50 LPM.

### 3.3. MMAD

**Figure 6:**
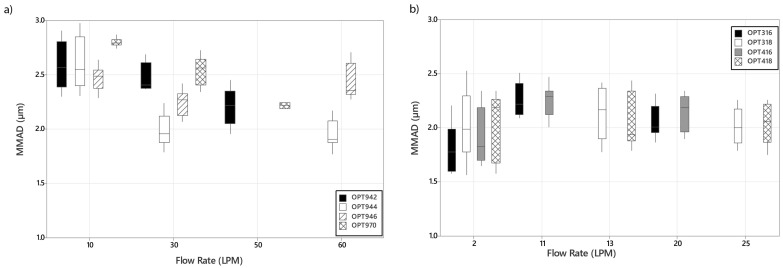
Plot of variations in mass median aerodynamic diameter (MMAD) (avg ± SD) with changes in nasal cannula and supplemental gas flow rate (LPM) for (**a**) adult and (**b**) infant and paediatric models.

Figure 6 presents the variation in MMAD (avg ± SD) with changes in interface, nasal cannula, and supplemental gas flow rate (LPM) in the (a) adult and (b) infant and paediatric interfaces. Geometric standard deviation (GSD) (avg ± SD) data are included in the supplementary data, Tables S5 and S6. Across the range of interface and nasal cannula in this study, the size of the aerosol droplet exiting the device was consistently smaller than that produced by the nebuliser, 4.11 ± 0.06 µm. In addition, in the adult devices shown in Figure 6a, the droplet diameter decreased with increasing flow rate from 2.59 ± 0.21 µm OPT942, 2.62 ± 0.24 µm OPT944, 2.47 ± 0.11 µm OPT946, and 2.81 ± 0.03 µm OPT970 at 10 LPM to 2.21 ± 0.17 µm OPT942 and 2.22 ± 0.03 µm OPT970 at 50 LPM and 1.96 ± 0.013 µm OPT944 and 2.45 ± 0.16 µm OPT946 at 60 LPM. Furthermore, at supplemental gas flows of 30 LPM and above, cannula size had a significant effect on MMAD, *p* ≤ 0.05, Table 5. With the paediatric nares as shown in Figure 6b, changes in nasal cannula size and flow rate had no significant effect on the MMAD, *p* ≥ 0.05, supplementary data Table S6. The opposite was found when changing to the infant nares. Increases in supplemental gas flow rate had a significant effect on the aerosol droplet diameter at the exit of the nares OPT316 and OPT 416, *p* < 0.05, Table 6.

## 4. Discussion

In this study, we completed a full performance characterisation of the Airvo2^TM^ HFO therapy system in combination with the Aerogen Solo^TM^ VMN. Variations in the emitted dose (%), tracheal dose (%), MMAD, and GSD with changes in supplemental gas flow rate, interface, and nasal cannula size were documented across the infant, paediatric, and adult ranges, as well as breathing profiles. 

In a study examining the parameters affecting the performance of high flow nasal therapy systems providing concurrent aerosol therapy, Bennett et al. found that to maximise the aerosol dose emitted, the supplemental gas flow rate should be kept low, the aerosol droplet size should be kept small, and the nebuliser should be positioned immediately after the humidification chamber [17]. Two of these factors, aerosol droplet size and nebuliser position, were kept constant throughout this study, while the nasal cannula size and supplemental gas flow rates were varied. 

As expected and in line with the numerous published studies that have examined trans-nasal aerosol delivery via HFO in the literature, supplemental gas flow rate was found to have an inverse relationship with the emitted and tracheal doses (%), irrespective of cannula size in the adult range, *p* ≤ 0.05 [18,33,34]. Cannula size was found to have a significant impact on both the emitted and tracheal doses (%) at supplemental gas flow rates of 30 LPM and below. Greater emitted and tracheal doses (%) were measured when the OPT946 was used at each flow rate considered as shown in Figure 4a, Figure 5a, and Tables S1 and S3 supplementary data, most likely due to the larger inner bore diameter of the OPT946 cannula, 6.0 mm ID versus 5.1 mm ID for the OPT944 and 4.2 mm ID for the OPT942. Smaller dimensions of the tubing and cannula components will hinder the exit of aerosol from the cannula during HFNO [35]. Similar trends to those reported here have been documented previously in the literature with this and other HFO therapy systems [4,17,18,34,35,36]. 

The data generated using the paediatric cannulas and settings follows the same trends as the adult data and other HFNO therapy paediatric studies in the literature [20,21]; increases in supplemental gas flow rate result in decreases in emitted and tracheal doses (%), as shown in Figure 4b and Figure 5b. Increasing cannula size from 2.9 mm ID OPT318 to 3.3 mm ID OPT418 had no significant effect on the emitted dose (%) below 25 LPM, *p* ≥ 0.05. However, at the lowest flow rate, 2 LPM, cannula size did have a significant effect on the tracheal dose (%), supplementary data Table S4, *p* ≤ 0.05. This is most likely due to losses in the head model. Interestingly, there were some key differences in the emitted and tracheal dose (%) data trends in the infant models OPT 316 and OPT416. While both decrease with increasing gas flow rate, 2–20 LPM, from 30.24–25.71% emitted and 8.11–4.45% tracheal OPT316 and from 31.64–25.46% emitted and 8.14–4.46% tracheal OPT416, both the emitted and tracheal doses (%) were significantly greater at 20 LPM than 11 LPM. In similar studies with the same infant head model SAINT, neither Wang et al. [20] nor Réminiac et al. [21] observed this phenomena. However, neither study extended the flow rate above 8 LPM. It is possible that at the higher gas flow rate, significant aerosol coalescence occurs and passes through the circuit to the collection filters. In a clinical setting, flows at this rate would most likely not be tolerated by the respective patient cohort and the findings here would serve to inform a clinical practice about reducing the gas flow rate during concurrent aerosol therapy in a HFNO therapy patient [37]. 

The opposite trends were found in the infant models OPT316 and OPT416, where above 2 LPM cannula ID had no significant effect on the emitted dose (%), *p* ≥ 0.05, supplementary data Table S2, and had no significant effect on the tracheal dose (%), *p* ≥ 0.05, supplementary data Table S4. Given that the infant cannula OPT316 and OPT416 had the smallest ID nares, 2.5 mm and 2.9 mm, respectively, and the smaller airway passages in the head model, this result is unsurprising. A recent scintigraphy study in infants reported values of 4.5 ± 2.2% deposition at a flow rate of 2 LPM using the same VMN [38]. The present study recorded values of 8.10 ± 0.85% with the OPT316 cannula and 8.14 ± 0.74% with the OPT416 cannula. The overestimation of our in vitro data is to be expected as the filters do not allow for the exhalation of aerosols that are not deposited. 

As noted in the introduction, to the best of the authors’ knowledge, there are neither in vivo nor in vitro studies that have detailed the aerosol delivery from the Airvo2^TM^ HFO therapy system where the bespoke nebuliser adapter and tracheostomy interface were used. Standard clinical practice incorporates a nebuliser into the respiratory circuit using a T-piece or tracheostomy collar. As such, there is no direct comparison possible between the data presented in this study with previous studies in the literature. Hence, the data presented in this study is the first to evaluate aerosol delivery via this means. Emitted and tracheal dose (%) data pertaining to the tracheostomy interface OPT970 follows the same trends as the nasal cannula data. Increases in supplemental gas rates cause a significant reduction in both the emitted and tracheal dose (%) measured. The emitted and tracheal doses (%) decrease from 9.38 ± 0.37% and 4.75 ± 0.53% at 10 LPM to 2.21 ± 0.31% and 0.40 ± 0.08% at 50 LPM. The low delivery efficiency compared to the nasal cannula—for example the OPT942 tracheal dose was 18.75 ± 0.83% at 10 LPM—is unsurprising given the design of the interface. The openings at the exit of the interface prior to the connection with the tracheostomy tube are a major source of aerosol loss. The use of a tracheostomy tube should result in a greater level of aerosol delivery as it bypasses the upper airways (naso/oropharynx and hypopharynx) where there is significant filtering and deposition of larger aerosol particles, increased resistance, and dead space [24].

MMAD is considered to be the optimal laboratory-based method to predict possible aerosol distribution within the lungs. It is the diameter that is larger than 50% of the aerosolised droplets and smaller than the remaining 50%, based on mass. In general, particles < 5 µm in diameter are considered suitable for inhaled drug delivery, with larger diameter droplets preferring to deposit in the upper airways [39,40]. The aerosol droplet sizes exiting the range of interfaces was consistently lower than that produced by the nebuliser, 4.11 ± 0.06 µm, Table 5 and Table 6. As the supplemental gas flow rate increased, the aerosol droplet diameter decreased significantly across the range of nasal and tracheostomy adult interfaces tested. This reduction is most likely caused by inertial impaction of the larger diameter aerosol droplets within the HFO therapy system and the cannula; thus, only the smaller diameter aerosol droplets can successfully transit through to the exit of the interfaces. Furthermore, at flow rates > 30 LPM, cannula diameter had a significant effect on the diameter of the aerosol droplets, *p* ≤ 0.05. These findings are consistent with those of both Bennett et al. [17] and Bhashyam et al. [41]. Cannula size had no significant effect on the aerosol droplet size exiting the paediatric and infant cannulas. Increases in supplemental gas flow and cannula diameter had no significant effect on the aerosol droplet size at the exit of the paediatric nares, *p* ≥ 0.05. This is most likely due to the filtering effect of the small diameter nares limiting the emission of the larger diameter particles, e.g., OPT 318 has a 2.9 mm ID and OPT418 has an ID of 3.3 mm. Contrasting aerosol droplet size trends were found when using the infant sized nares; as supplemental gas flow increased from 2–20 LPM, the aerosol droplet size increased. This was also reported in studies performed by Perry et al. [34] and Reminiac et al. [21]. The infant nares OPT 316 and OPT 416 have smaller internal diameters of 2.5 mm ID OPT316 and 2.9 mm ID OPT416, respectively, and should, in theory, have the smallest aerosol droplet diameters exiting the nares, particularly at the higher flow rates. The increase in aerosol droplet diameter with increasing flow rate may occur due to increased levels of rained-out humidity that occur within the smaller tubing and nares which would artificially increase the droplet size measurements. 

There are several limitations to this study. In line with international test standards, e.g., ISO27427 and the FDA guidance, tracheal doses (%) were assessed with breathing patterns associated with simulated healthy adults, infants, and paediatrics. However, recipients of HFO therapy would, most likely, not present with such breathing patterns. Breath pattern has been shown to affect the delivered dose, and as such, whilst this can be considered a gold standard data set describing the performance of the Airvo2 and Aerogen Solo combination, differences may be expected in the clinical setting. There are a number of different designs of nasal cannula in use in the critical and home care setting, including the asymmetrical nasal cannula. These new designs offer greater patient comfort and airway CO_2_ removal. However, there are few studies that have examined their effect on aerosol delivery. This study was completed using atmospheric air containing 21% oxygen. However, in certain clinical situations, blends are used to deliver a higher oxygen concentration. Studies are required to determine the effects, if any, of higher oxygen concentrations and other blended carrier gasses on aerosol delivery to different patient types and cohorts. 

## 5. Conclusions

This study entailed a full system characterisation of the Airvo2^TM^ HFO therapy system used concurrently with the Aerogen Solo^TM^ VMN and associated controller to deliver aerosol therapy. Nasal cannulas and tracheostomy interfaces were used to deliver the aerosol. The effects of gas flow rate and nasal cannula size across the adult, infant, and paediatric product range on the emitted and tracheal doses (%) and aerosol droplet diameter, MMAD, were considered. To maximise aerosol delivery, gas flow rates should be kept to a minimum when both interface types are used in the adult models. Furthermore, larger diameter nares should be used with adults receiving aerosol therapy via nasal cannula. In infants and paediatrics, gas flow rates should also be kept low, however, cannula size was not found to have a significant effect on the dose of aerosol available at the tracheal level (%) in infants but was at very low flow rates in paediatrics. Gas flow rate was found to have the greatest influence on the diameter of the aerosol droplets exiting the interfaces, with increases in gas flow resulting in a reduction in the aerosol droplet diameter. Although this study was conducted in vitro, it should be of significant benefit and interest to clinicians when planning treatment strategies to maximise aerosol delivery to patients receiving HFO therapy.

## Figures and Tables

**Table 1:** Details of the nasal cannulas included in the study.

Cannula Reference	Size	Approved Gas Flow Rate Range (LPM)
OPT942	Small Adult Cannula	10–50
OPT944	Medium Adult Cannula	10–60
OPT946	Large Adult Cannula	10–60
OPT316	Infant Nasal Cannula	2–20
OPT318	Paediatric Nasal Cannula	2–25
OPT416	Infant Nasal Cannula	2–20
OPT418	Paediatric Nasal Cannula	2–25
OPT970	Tracheostomy Interface	10–60

**Table 2:** Emitted and tracheal dose test combinations using adult nasal cannula.

Head Model	Paediatric	Adult	Adult	Adult
Gas Flow (LPM)	OPT942 * (S)	OPT944 (M)	OPT946 (L)	OPT970
10	✓	✓	✓	✓
30	✓	✓	✓	✓
50	✓	Not tested	Not tested	✓
60	Not tested	✓	✓	Not tested

* OPT942 (small adult cannula) were too small to be used with the adult head model when following manufacturer’s cannula sizing/fitting instructions; therefore, the paediatric head model was used.

**Table 3:** Emitted and tracheal dose test combinations using infant and paediatric nasal cannula.

Head Model	Infant	Paediatric	Infant	Paediatric
Gas Flow (LPM)	OPT316	OPT318	OPT416	OPT418
2	✓	✓	✓	✓
11	✓	Not tested	✓	Not tested
13	Not tested	✓	Not tested	✓
20	✓	Not tested	✓	Not tested
25	Not tested	✓	Not tested	✓

**Table 4:** Breathing patterns used to simulate the different simulated patient groups (ISO27427: 2019) [30].

Head Model	Tidal Volume (mL)	Breaths per Minute	I/E Ratio
Adult	500	15	1:1
Paediatric	155	25	1:2
Infant	50	30	1:3

**Table 5:** Variations in mass median aerodynamic diameter (MMAD) (µm) (avg ± SD) with changes in gas flow rate (LPM) and nasal cannula size for adult devices; *p* < 0.05 indicates significance.

Gas Flow (LPM)	OPT942	OPT944	OPT946	*p*-Value	OPT970
10	2.59 ± 0.21	2.62 ± 0.24	2.47 ± 0.11	0.247	2.81 ± 0.03
30	2.50 ± 0.13	2.00 ± 0.15	2.24 ± 0.12	0.000	2.54 ± 0.15
50	2.21 ± 0.17	Not tested	Not tested	-	2.22 ± 0.03
60	Not tested	1.96 ± 0.13	2.45 ± 0.16	0.000	Not tested
*p*-value	0.000	0.000	0.002		0.001

**Table 6:** Variations in mass median aerodynamic diameter (MMAD) (µm) (avg ± SD) with changes in gas flow rate (LPM) and nasal cannula size for infant and paediatric devices; *p* < 0.05 indicates significance.

Gas Flow (LPM)	OPT316	OPT416	*p*-Value	OPT318	OPT418	*p*-Value
2	1.80 ± 0.23	1.93 ± 0.26	0.089	2.02 ± 0.31	2.04 ± 0.31	0.740
11	2.27 ± 0.16	2.24 ± 0.14	0.444	Not tested		-
13	Not tested		-	2.14 ± 0.24	2.06 ± 0.25	0.119
20	2.06 ± 0.15	2.14 ± 0.17	0.091	Not tested		-
25	Not tested		-	2.01 ± 0.17	2.03 ± 0.19	0.232
*p*-value	0.000	0.007		0.491	0.975	

## Data Availability

The data presented in this study are available in this article (and Supplementary Materials).

## Supplementary Materials

The following supporting information can be downloaded at: https://www.mdpi.com/article/10.3390/pharmaceutics16040565/s1, Table S1: Variations in emitted dose (%) (avg ± SD) with changes in gas flow rate (LPM), interface and nasal cannula size for adult devices, *p* < 0.05 indicates significance; Table S2: Variations in emitted dose (%) (avg ± SD) with changes in gas flow rate (LPM) and nasal cannula size for paediatric and infant devices, *p* < 0.05 indicates significance; Table S3: Variations in tracheal dose (%) (avg ± SD) with changes in gas flow rate (LPM), interface and nasal cannula size for adult devices, *p* < 0.05 indicates significance; Table S4: Variations in tracheal dose (%) (avg ± SD) with changes in gas flow rate (LPM) and nasal cannula size for paediatric and infant devices, *p* < 0.05 indicates significance; Table S5: Variations in geometric standard deviation (GSD) (µm) (avg ± SD) with changes in gas flow rate (LPM), interface and nasal cannula size for adult devices, *p* < 0.05 indicates significance; Table S6: Variations in geometric standard deviation (GSD) (µm) (avg ± SD) with changes in gas flow rate (LPM) and nasal cannula size for paediatric and infant devices, *p* < 0.05 indicates significance.
