# Supplementary material for: Performance Characterisation of the Airvo2TM Nebuliser Adapter in Combination with the Aerogen SoloTM Vibrating Mesh Nebuliser for in Line Aerosol Therapy during High Flow Nasal Oxygen Therapy
Source: Pharmaceutics. 2024 Apr 20;16(4):565. doi: 10.3390/pharmaceutics16040565 (PMC11053618; doi:10.3390/pharmaceutics16040565)
Supplement: Supplementary file 1 [file pharmaceutics-16-00565-s001.zip › pharmaceutics-2953280-supplementary.pdf]

**Supplementary Materials:**

## Emitted dose data

**Table S1.** Variations in emitted dose (%) (avg ± SD) with changes in gas flow rate (LPM), interface and nasal cannula size for adult devices, $p < 0.05$ indicates significance.

| Gas Flow (LPM) | OPT942 | OPT944 | OPT946 | P-value | OPT970 |
|---|---|---|---|---|---|
| **10** | 37.78 ± 2.83 | 39.45 ± 3.08 | 50.87 ± 2.74 | 0.000 | 9.38 ± 0.37 |
| **30** | 25.68 ± 1.66 | 36.98 ± 2.85 | 35.74 ± 3.88 | 0.000 | 7.51 ± 0.72 |
| **50** | 9.96 ± 1.59 | Not tested | Not tested | - | 2.21 ± 0.31 |
| **60** | Not tested | 11.81 ± 1.36 | 11.83 ± 1.55 | 0.984 | Not tested |
| **P-value** | 0.000 | 0.000 | 0.000 | - | 0.000 |

**Table S2.** Variations in emitted dose (%) (avg ± SD) with changes in gas flow rate (LPM) and nasal cannula size for paediatric and infant devices, $p < 0.05$ indicates significance.

| Gas Flow (LPM) | OPT316 | OPT416 | P-value | OPT318 | OPT418 | P-value |
|---|---|---|---|---|---|---|
| **2** | 30.24 ± 1.37 | 31.64 ± 1.67 | 0.006 | 30.89 ± 1.27 | 31.65 ± 1.88 | 0.119 |
| **11** | 24.87 ± 0.65 | 25.16 ± 0.83 | 0.081 | Not tested | Not tested | - |
| **13** | Not tested | Not tested | - | 25.71 ± 0.92 | 25.46 ± 0.78 | 0.720 |
| **20** | 25.71 ± 0.50 | 25.46 ± 1.23 | 0.458 | Not tested | Not tested | - |
| **25** | Not tested | Not tested | - | 24.04 ± 1.10 | 24.68 ± 1.04 | 0.000 |
| **P-value** | 0.000 | 0.000 | | 0.000 | 0.000 | |

## Tracheal dose data

**Table S3.** Variations in tracheal dose (%) (avg ± SD) with changes in gas flow rate (LPM), interface and nasal cannula size for adult devices, $p < 0.05$ indicates significance.

| Gas Flow (LPM) | OPT942 | OPT944 | OPT946 | P-value | OPT970 |
|---|---|---|---|---|---|
| **10** | 18.75 ± 0.83 | 19.32 ± 0.97 | 21.78 ± 0.66 | 0.000 | 4.75 ± 0.53 |
| **30** | 14.43 ± 0.41 | 5.89 ± 0.33 | 14.28 ± 0.49 | 0.000 | 2.84 ± 0.40 |
| **50** | 5.22 ± 0.20 | Not tested | Not tested | - | 0.40 ± 0.08 |
| **60** | Not tested | 1.77 ± 0.26 | 1.68 ± 0.27 | 0.139 | Not tested |
| **P-value** | 0.000 | 0.000 | 0.000 | - | 0.000 |

**Table S4.** Variations in tracheal dose (%) (avg ± SD) with changes in gas flow rate (LPM) and nasal cannula size for paediatric and infant devices, p < 0.05 indicates significance.

| Gas Flow (LPM) | OPT316 | OPT416 | P-value | OPT318 | OPT418 | P-value |
|---|---|---|---|---|---|---|
| **2** | 8.10 ± 0.85 | 8.14 ± 0.74 | 0.581 | 8.14 ± 0.74 | 9.78 ± 0.46 | 0.000 |
| **11** | 3.88 ± 0.36 | 3.81 ± 0.29 | 0.206 | Not tested | Not tested | - |
| **13** | Not tested | Not tested | - | 4.88 ± 0.45 | 4.94 ± 0.26 | 0.522 |
| **20** | 4.45 ± 0.16 | 4.46 ± 0.18 | 0.713 | Not tested | Not tested | - |
| **25** | Not tested | Not tested | - | 1.93 ± 0.35 | 1.89 ± 0.33 | 0.449 |
| **P-value** | 0.000 | 0.000 | | 0.000 | 0.000 | |

## GSD data

**Table S5.** Variations in geometric standard deviation (GSD) (µm) (avg ± SD) with changes in gas flow rate (LPM), interface and nasal cannula size for adult devices, p < 0.05 indicates significance.

| Gas Flow (LPM) | OPT942 | OPT944 | OPT946 | P-value | OPT970 |
|---|---|---|---|---|---|
| **10** | 1.61 ± 0.04 | 1.68 ± 0.07 | 1.69 ± 0.06 | 0.015 | 1.93 ± 0.10 |
| **30** | 1.64 ± 0.05 | 1.90 ± 0.12 | 1.81 ± 0.08 | 0.003 | 2.50 ± 0.76 |
| **50** | 1.70 ± 0.14 | Not tested | Not tested | - | 2.12 ± 0.38 |
| **60** | Not tested | 1.78 ± 0.06 | 1.93 ± 0.08 | 0.003 | Not tested |
| **P-value** | 0.106 | 0.009 | 0.003 | | 0.406 |

**Table S6.** Variations in geometric standard deviation (GSD) (µm) (avg ± SD) with changes in gas flow rate (LPM) and nasal cannula size for paediatric and infant devices, p < 0.05 indicates significance.

| Gas Flow (LPM) | OPT316 | OPT416 | P-value | OPT318 | OPT418 | P-value |
|---|---|---|---|---|---|---|
| **2** | 1.64 ± 0.07 | 1.68 ± 0.11 | 0.335 | 1.72 ± 0.09 | 1.68 ± 0.13 | 0.542 |
| **11** | 1.77 ± 0.19 | 1.71 ± 0.09 | 0.366 | Not tested | | - |
| **13** | Not tested | | - | 1.62 ± 0.05 | 1.62 ± 0.06 | 0.920 |
| **20** | 1.76 ± 0.16 | 1.62 ± 0.09 | 0.048 | Not tested | | - |
| **25** | Not tested | | - | 1.61 ± 0.08 | 1.86 ± 0.10 | 0.002 |
| **P-value** | 0.139 | 0.110 | | 0.012 | 0.000 | |
